# Efficacy of Bidirectional Paclitaxel plus Capecitabine or Nilotinib for Peritoneal Carcinomatosis: A Single Institution Analysis of Two Phase II Clinical Trials

**DOI:** 10.1245/s10434-025-18967-2

**Published:** 2026-01-16

**Authors:** Amber F. Gallanis, Shruthi R. Perati, Stephanie N. Canady, Monica Epstein, Nancy Moore, Audra A. Satterwhite, Yvonne Mallory, Diane Ahn, Cassidy Bowden, Silvia Figueiroa, John J. Lee, Andre de Souza, Sarah Shin, Jibran Ahmed, Molly A. Sullivan, Stacy R. Joyce, Jonathan M. Hernandez, Alice Chen, Christine C. Alewine, Jeremy L. Davis, Andrew M. Blakely

**Affiliations:** 1 Surgical Oncology Program, Center for Cancer Research, National Cancer Institute, National Institutes of Health, Bethesda, MD USA; 2 Developmental Therapeutics Clinic, Division of Cancer Treatment and Diagnosis, National Cancer Institute, National Institutes of Health, Bethesda, MD USA; 3 Clinical Center, National Institutes of Health, Bethesda, MD USA; 4 Dartmouth Cancer Center, Geisel School of Medicine at Dartmouth, Lebanon, NH USA; 5 Department of Surgery, University of Maryland School of Medicine, Greenebaum Comprehensive Cancer Center, Baltimore, MD USA

**Keywords:** Peritoneal carcinomatosis, Intraperitoneal chemotherapy, Bidirectional therapy, Gastric adenocarcinoma, Appendiceal adenocarcinoma

## Abstract

**Background:**

The efficacy of locoregional therapy with intraperitoneal (IP) drug delivery plus systemic chemotherapy for peritoneal carcinomatosis is understudied. We investigated progression-free survival (PFS) and overall survival (OS) in patients with peritoneal carcinomatosis from gastric, appendiceal, or small bowel adenocarcinoma who received intravenous (IV) and IP paclitaxel plus capecitabine or nilotinib.

**Methods:**

Two separate single-institution phase II clinical trials evaluating IP and IV paclitaxel therapy plus capecitabine or nilotinib for peritoneal carcinomatosis were analyzed. Enrolled patients with peritoneal-only metastatic gastric cancer received IP and IV paclitaxel plus capecitabine. Participants with peritoneal carcinomatosis from appendiceal, gastric, or small bowel adenocarcinoma received IP and IV paclitaxel plus nilotinib.

**Results:**

Twelve patients with a median age of 46 years (range 38–64) received bidirectional paclitaxel plus capecitabine. Median overall PFS and OS was 5.3 months (95% confidence interval [CI] 1.5–13.3) and 12.5 months (95% CI 4.7–14.7), respectively. Seven patients with peritoneal carcinomatosis from appendiceal, gastric, or small bowel adenocarcinoma with a median age 59 years (range 46–69) received bidirectional paclitaxel plus nilotinib. Median PFS and OS was 3.6 months (range 2.6–6.6) and 8.3 months (range 2.8–10.2), respectively, for those receiving bidirectional paclitaxel plus nilotinib. Adverse events (AEs) were common; grade 3–5 AEs occurred in 90.1% (10/11) of participants receiving IP/IV paclitaxel plus capecitabine and 100% (7/7) of patients receiving IP/IV paclitaxel plus nilotinib. There was no extra-peritoneal disease progression, suggesting tumor confinement among all participants.

**Conclusions:**

Bidirectional paclitaxel-based chemotherapy plus capecitabine may delay progression of gastric adenocarcinoma with peritoneal-only metastasis. Bidirectional paclitaxel-based chemotherapy plus nilotinib was associated with mostly stable peritoneal disease in this small, heterogenous cohort. Bidirectional paclitaxel combinations are feasible and may have a role in therapy for disease stabilization in individuals with peritoneal carcinomatosis.

Peritoneal carcinomatosis is characterized by aggressive disease with a dismal prognosis and poor outcomes. Early peritoneal dissemination commonly occurs and is present at diagnosis in approximately 23–43% of gastric and 75–80% of ovarian cancers.^1–5^ Systemic chemotherapy is the mainstay of treatment; however, recent clinical trials have demonstrated the efficacy of cytoreductive surgery (CRS) with or without hyperthermic intraperitoneal (IP) chemotherapy (HIPEC) for the treatment of peritoneal carcinomatosis from appendiceal, ovarian, colorectal, and gastric cancers.^6–12^ Previous literature suggests completeness of cytoreduction is the strongest predictor of improved survival in patients with peritoneal carcinomatosis, yet only a proportion of patients with peritoneal-only metastatic disease are eligible for CRS with or without HIPEC.^13,14^ Additionally, CRS is highly invasive and imparts significant morbidity and mortality.^15,16^

Currently, regional therapies for peritoneal carcinomatosis are investigational. Previous studies have demonstrated that bidirectional chemotherapy or catheter-based IP chemotherapy without cytoreduction, in combination with systemic chemotherapy, is safe and generally well-tolerated.^17,18^ Long dwell times and repeated dosing of IP chemotherapy promotes tumor penetration and higher peritoneal cavity drug concentrations than systemic therapy.^19^ Taxanes represent ideal IP chemotherapeutic agents because of their hydrophobic properties and high molecular weight, which allows for prolonged retention in the peritoneal space.^18–20^ Repeated IP taxane administration also rarely causes peritoneal fibrosis, making surgical resection technically feasible after iterative IP paclitaxel administration.^19,21^

To enhance the antitumor effect of bidirectional taxanes and to provide another pathway for cytotoxicity, the addition of a systemic agent is often desired. Fluorouracil has been a frequently employed cytotoxic agent for a multitude of cancers, both in combination for active treatment and as monotherapy in the adjuvant setting. Previous phase III clinical trials have demonstrated improved overall survival (OS) in patients with advanced gastric cancer treated with the addition of a taxane to cisplatin and fluorouracil compared with cisplatin and fluorouracil alone, supporting the use of capecitabine in bidirectional paclitaxel treatment paradigms.^22^ Alternatively, a recent phase I study of combination paclitaxel and nilotinib demonstrated overall safety and antitumor activity with confirmed partial responses in individuals with ovarian granulosa cell tumors and endometrial carcinoma.^23^ However, there are limited clinical trials for IP/IV paclitaxel combinations, and the efficacy of IP/IV paclitaxel plus capecitabine or nilotinib for treatment of peritoneal carcinomatosis is currently unknown.

The aims of our studies were to determine the role of bidirectional paclitaxel-based chemotherapy plus capecitabine or nilotinib in unresectable, peritoneal-only, metastatic appendiceal, small bowel, gastric, or gastroesophageal junction cancers. Our primary outcomes were progression-free survival (PFS) and downstaging disease burden to potential resectability based on peritoneal cancer index (PCI) score. Secondary outcomes included OS, extra-peritoneal disease progression, and safety evaluation of IP/IV paclitaxel plus capecitabine or nilotinib.

## Methods

### Participant Eligibility

**Fig. 1:**
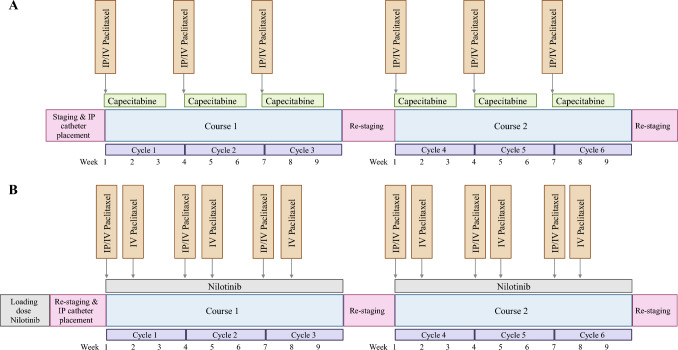
(A) Intraperitoneal (IP)/intravenous (IV) paclitaxel plus capecitabine study design and (B) IP/IV paclitaxel plus nilotinib study design for enrolled patients. Created with biorender.com

Two separate phase II, non-randomized, open-label, single-arm clinical trials at the National Institutes of Health (NIH) Clinical Center evaluating IV and IP paclitaxel therapy plus capecitabine (NCT04034251) or nilotinib (NCT05185947) for peritoneal carcinomatosis were analyzed. Patients with gastric or gastroesophageal junction adenocarcinoma with peritoneal-only metastases were eligible to enroll in clinical trial NCT04034251 and receive bidirectional paclitaxel therapy (IP paclitaxel 20 mg/m^2^ or 60 mg/m^2^ and IV paclitaxel 80 mg/m^2^) in combination with oral capecitabine (875 mg/m^2^). Alternatively, patients with peritoneal carcinomatosis from colorectal, appendiceal, small bowel, gastric, cholangiocarcinoma, breast, ovarian, or other gynecologic primary cancers were eligible to enroll in clinical trial NCT05185947 and receive bidirectional paclitaxel therapy (IP paclitaxel 60 mg/m^2^ and 80 mg/m^2^ and IV paclitaxel 60 mg/m^2^ and 80 mg/m^2^) plus oral nilotinib (300 mg twice daily) (Fig. 1). Both studies were approved by the institutional review board of the NCI, NIH. All patients provided written consent for treatment. All enrolled participants had histologically or cytologically confirmed peritoneal carcinomatosis identified radiographically or on staging laparoscopy. Additional eligibility criteria for both clinical trials included age ≥ 18 years, Eastern Cooperative Oncology Group performance status ≤ 2, adequate organ and bone marrow function, and medical fitness for laparoscopy and systemic chemotherapy. Exclusion criteria included presence of disseminated extra-peritoneal metastasis, > 3 L of ascites present at initial laparoscopy, previous disease progression on paclitaxel, and previous regional therapy within past 6 months.

### Bidirectional Paclitaxel and Capecitabine Study Design

All participants underwent imaging studies (computed tomography and/or positron emission tomography) for preliminary staging. During diagnostic laparoscopy, a peritoneal access catheter (BardPort^®^, Bard Access System, Salt Lake City, UT, USA) was placed and bidirectional paclitaxel plus oral capecitabine was initiated (1A). Once it was established that the initial IP paclitaxel dose (20 mg/m^2^) was tolerated and could be safely administered to patients, the dose was increased to 60 mg/m^2^ for the remaining patients enrolled in the study. Each 3-week treatment cycle consisted of one dose of IP paclitaxel (20 mg/m^2^ or 60 mg/m^2^) plus one dose of IV paclitaxel (80 mg/m^2^) on cycle 1 day 1, followed by 14 days of capecitabine 875 mg/m^2^ twice daily, and a 7-day treatment-free interval (Fig. 1A).

### Bidirectional Paclitaxel and Nilotinib Study Design

Cross-sectional imaging (computed tomography and/or positron emission tomography) and screening laparoscopy were performed by unblinded evaluators to confirm patient eligibility. Participants received a loading dose of nilotinib 300 mg twice daily within 6 weeks of initial screening laparoscopy. At 4 days after patients received the initial dosing of nilotinib (i.e., days -4, -3, -2, -1), a second laparoscopy with peritoneal catheter placement (BardPort^®^, Bard Access System, Salt Lake City, UT, USA) (day 0) was performed. IP paclitaxel (60 mg/m^2^) was administered the day after IP catheter placement (cycle 1 day 1) and again during the first week of each 3-week treatment cycle for cycles 1–3 (Fig. 1B). For cycles 4–6, the IP paclitaxel dose could be increased to 80 mg/m^2^, if well-tolerated. The first dose of IV paclitaxel was 60 mg/m^2^ to ensure patient tolerability, administered on cycle 1 day 2; thereafter, it was increased to 80 mg/m^2^ and administered on cycle 1 day 8 and on day 1 and 8 of each subsequent treatment cycle. Oral nilotinib 300 mg was administered twice daily during the 18-week treatment course, except on mornings of diagnostic laparoscopy. Patients who completed 6 cycles of treatment and had stable disease had the option of continuing with IV paclitaxel and oral nilotinib for up to 1 additional year. Diagnostic laparoscopy with peritoneal biopsy sampling was performed upon completion of 3 cycles and of 6 cycles of treatment for restaging of disease.

In both clinical trials, initial laparoscopy was performed to determine PCI scoring and extent of peritoneal carcinomatosis.^24^ Patients with an objective response to treatment, defined as a reduction in PCI of ≥ 4 from baseline, or stable disease, defined as PCI within 2–3 points from baseline, were continued on study and planned to undergo another 3 cycles (9 weeks) of bidirectional treatment. Patients with progressive disease, defined as an increase in PCI by >4 from baseline, were taken off study.

### Study Endpoints

The primary study endpoints were PFS for the bidirectional paclitaxel plus capecitabine trial and downstaging of peritoneal disease burden to resectability for the bidirectional paclitaxel plus nilotinib trial. Secondary outcome measures included OS, extra-peritoneal disease progression, and the frequency of treatment-related adverse events (AEs). AEs were categorized by grade according to Common Terminology Criteria for Adverse Events (CTCAE) version 5.0.^25^ Individuals receiving paclitaxel and capecitabine who experienced grade 3 or higher AEs had treatment delay until toxicity resolved. Those receiving paclitaxel and nilotinib who experienced AEs had a maximum of two dose reductions before the participant was taken off treatment.

### Statistical Analysis

Median PFS and OS was calculated with a 95% confidence interval (CI). All data were analyzed using GraphPad Prism version 9.3.1 (San Diego, CA, USA), and probabilities of recurrence and survival were estimated using the Kaplan–Meier method.

## Results

### Bidirectional Paclitaxel and Capecitabine

#### Patient Characteristics

**Fig. 2:**
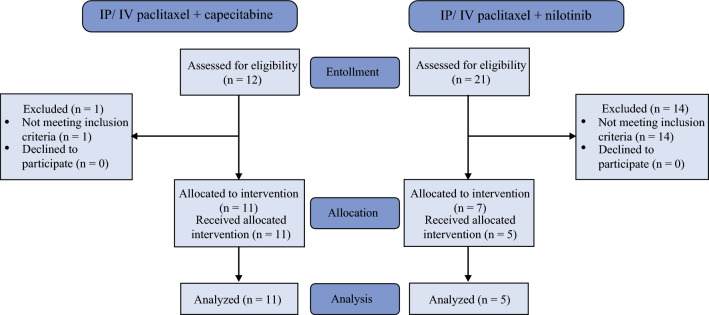
Consolidated Standards of Reporting Trials (CONSORT) flow diagram for bidirectional paclitaxel plus capecitabine or nilotinib clinical trials. IP, intraperitoneal; IV, intravenous

In total, 12 patients with peritoneal-only metastatic gastric adenocarcinoma or gastroesophageal junction adenocarcinoma were enrolled from January 21, 2020, through August 25, 2022, in a phase II, single-arm, open-label clinical trial at the NIH Clinical Center to receive bidirectional paclitaxel and capecitabine (Fig. 2). One patient developed extra-peritoneal disease after study enrollment, prior to IP catheter placement, and was taken off study. In total, there were six (55%) males and five (45%) females, comprised mostly of white individuals (73%; 8/11), with a median age of 47 years (range 38–64) at study enrollment (Table 1). Most individuals had their primary tumor location in the stomach (91%; 10/11), and one patient had a gastroesophageal junction primary tumor (9%; 1/11). Only one patient underwent previous oncologic resection with total gastrectomy and lymphadenectomy for pT4N0 gastric cancer. All patients had prior doublet or triplet systemic chemotherapy with a median of two (range 1–3) lines of therapy per patient (Table 2). The median baseline PCI in the low-dose (20 mg/m^2^) IP paclitaxel group (n = 4) was 16 (range 11–20) compared with 13 (range 10–15) in the high-dose (60 mg/m^2^) IP paclitaxel group (n=7). Among all study participants, the median baseline PCI was 13 (range 10–20), with median restaging PCI of 13 (range 11–22) and 12 (range 8–13) after completion of treatment course one and two, respectively. The median number of cycles completed for all study participants was three (range 2–6) (Table 1).

**Table 1:** Demographic and oncologic patient characteristics

Characteristics	IP/IV paclitaxel plus capecitabine			IP/IV paclitaxel plus nilotinib
	IP paclitaxel (20 mg/m^2^) (n=4)	IP paclitaxel (60 mg/m^2^) (n=7)	Total (n=11)	Total (n=7)^a^
Age at enrollment, years	46 (38–64)	52 (38–63)	47 (38–64)	59 (46–69)
Sex				
Male	1 (25)	5 (71)	6 (55)	6 (86)
Female	3 (75)	2 (29)	5 (45)	1 (14)
Race				
White	2 (50)	6 (86)	8 (73)	5 (71)
Hispanic	1 (25)	1 (14)	2 (18)	–
Asian	1 (25)	–	1 (9)	2 (29)
Location of primary tumor				
Appendiceal	–	–	–	3 (43)
GE junction	1 (25)	–	1 (9)	–
Gastric adenocarcinoma				
Cardia	1 (25)	1 (14)	2 (18)	–
Fundus	1 (25)	1 (14)	2 (18)	1 (14)
Body	1 (25)	3 (43)	4 (36)	2 (29)
Antrum	–	2 (29)	2 (18)	–
Small bowel adenocarcinoma	–	–	–	1 (14)
Previous oncologic resection	–	1 (14)	1 (9)	1 (14)
PCI upon enrollment	16 (11–20)	13 (10–15)	13 (10–20)	16 (13–24)
Cycles completed	5 (2–6)	3 (2–6)	3 (2–6)	4 (3–7)

Data are presented as median (range) or n (%) unless otherwise indicated

^a^Includes two patients taken off study prior to completion of cycle 1

GE, gastroesophageal; IP/IV, intraperitoneal/intravenous; PCI, Peritoneal Carcinomatosis Index

**Table 2:** Prior systemic chemotherapy and tumor response

IP/IV paclitaxel plus capecitabine							
Patient	Preoperative systemic therapy	PCI upon enrollment	Re–staging PCI #2	Re–staging PCI #3	Tumor response	Time to disease progression (mo)	Overall survival (mo)
1	FLOT	11	11	8	SD	6^a^ (off study for TG and HIPEC)	12.7
2	FLOT	20	–	–	PD	1.4	4.7
3	FLOT, capecitabine	20	22	–	SD	3.2^a^ (off study for IL–15 therapy)	8.9
4	FOLFOX, nivolumab, FOLFIRI	12	13	13	SD	5.3^a^ (off study for TG and HIPEC)	12.6
5	FOLFOX, capecitabine	13	14	–	SD	5.8^a^ (off study for targeted claudin therapy)	14.9
6	FLOT	10	15	–	PD	2.3^b^	11.5
7	FOLFOX, nivolumab	12	–	–	PD	1.5	4.1
8	FOLFOX, paclitaxel/ ramucirumab	12	12	11	SD	5.3^a^ (off study for alternative systemic therapy)	16.5
9	FOLFOX	13	–	–	PD	2.1	9.1
10	FOLFOX, nivolumab	15	13	13	SD	13.5	17.9
11	FOLFOX	13	13	–	SD	5.4^a^ (off study for laparoscopic HIPEC therapy)	Alive
*IP/IV paclitaxel plus nilotinib*							
1	FOLFOX	15	9	–	OR	5.8	10.2
2	FOLFOX, FOLFOXIRI/avastin, FOLFIRI/panitumumab	20	18	–	SD	2.7	2.7
3	Docetaxel/cisplatin/5–FU, FOLFOX/nilotinib	20	20	–	SD	3.7	3.7
4	FLOT	16	16	19	SD	6.5	8.3
5	FOLFOX with nivolumab, 5–FU monotherapy, FOLFIRI, paclitaxel	13	10	–	SD	3.5	Alive

FLOT, 5-fluorouracil, leucovorin, oxaliplatin, and docetaxel; FOLFIRI, 5-fluorouracil, leucovorin, irinotecan; FOLFOX, 5-fluorouracil, leucovorin, oxaliplatin; FOLFOXIRI, 5-fluorouracil, leucovorin, oxaliplatin, irinotecan; 5-FU, 5-fluorouracil; HIPEC, hyperthermic intraperitoneal chemotherapy; IL, interleukin; IP/IV, intraperitoneal/intravenous; OR, objective response; PCI, Peritoneal Carcinomatosis Index; PD, progressive disease; mo, months; SD, stable disease; TG, total gastrectomy

^a^Moved off study to pursue alternative therapies

^b^Missed cycles due to COVID-19 infection

### Clinical Outcomes and Survival Characteristics

**Fig. 3:**
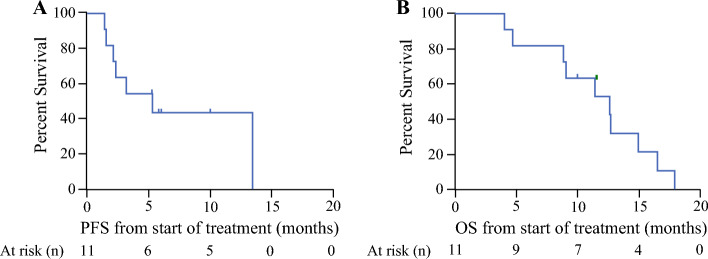
Kaplan–Meier curves for (A) clinical progression-free survival (PFS) and (B) overall survival (OS) from start of intraperitoneal/intravenous paclitaxel plus capecitabine treatment in months

The majority (64%; 7/11) of patients receiving IP/IV paclitaxel and capecitabine had stable disease, defined as PCI within 2 of baseline, during the trial period. Four (36%) of 11 patients exhibited disease progression after the first treatment course and were taken off study (Table 2). Multiple participants with stable disease were eventually taken off study to pursue alternative therapies such as targeted claudin therapy, interleukin (IL)-15, and CRS with HIPEC (Table 2). Frequency of tumor objective histopathologic response to therapy was not reported, as there was no evidence of objective pathologic response based on peritoneal biopsies collected during restaging laparoscopy. Importantly, no patients developed extra-peritoneal disease progression during the study period. Median PFS was 4.2 months (95% CI 1.4–not achieved) and 13.3 months (95% CI 1.5–13.3; *p* = 0.5) for the IP paclitaxel 20 mg/m^2^ and 60 mg/m^2^ groups, respectively. Median OS was 10.6 months (95% CI 4.7–12.5) for those receiving low-dose IP paclitaxel compared with 14.7 months (95% CI 4.0–17.7; *p* = 0.18) in the high-dose IP paclitaxel group. Combined PFS and OS among all individuals receiving bidirectional paclitaxel and capecitabine was 5.3 months (95% CI 1.5–13.3) and 12.5 months (95% CI 4.7–14.7), respectively (Fig. 3).

### Safety and Morbidity

The most reported grade 3–5 AE per patient was neutropenia (54.5%; 6/11), abdominal pain (27%; 3/11), and anemia (18.2%; 2/11) (Table 3). One patient developed an IP port site infection that was treated with IV antibiotics, and one patient was admitted for treatment-related abdominal pain and diagnosed with neutropenic enterocolitis. Grade 1 and 2 AEs occurred in all individuals on study (100%; 11/11). The most frequent grade 1–2 AEs were leukopenia (45.5%; 5/11), neuropathy (27.3%; 3/11), diarrhea (18.2%; 2/11), nausea (18.2%; 2/11), and lymphopenia (18.2; 2/11) (Table 3). All-cause mortality of the study cohort was 91% (10/11) at the time of study closure.

**Table 3:** Highest-grade adverse events per patient

CTCAE term	IP/IV paclitaxel plus capecitabine		IP/IV paclitaxel plus nilotinib	
	Grade 1–2 per patient n/11 (%)	Grade 3–5 per patient n/11 (%)	Grade 1–2 per patient n/7 (%)	Grade 3–5 per patient n/7 (%)
*Gastrointestinal*				
Abdominal pain	1 (9.1)	3 (27.3)	1 (14.3)	1 (14.3)
Diarrhea	2 (18.2)	1 (9.1)	–	1 (14.3)
Peritoneal infection	–	–	–	1 (14.3)
Vomiting	–	–	2 (28.6)	–
Anorexia	–	–	1 (14.3)	–
Dehydration	–	–	1 (14.3)	–
Gastroesophageal reflux disease	–	–	1 (14.3)	–
Dysphagia	1 (9.1)	–	–	–
Nausea	2 (18.2)	–	1 (14.3)	–
Constipation	–	–	1 (14.3)	–
Mucositis	1 (9.1)	–	–	–
Abdominal infection	1 (9.1)	1 (9.1)^a^	–	–
Enterocolitis infectious	–	1 (9.1)	–	–
*Hematologic*				
Thromboembolic event	–	–	1 (14.3)	1 (14.3)
Anemia	1 (9.1)	2 (18.2)	–	2 (28.6)
White blood cell decreased	5 (45.5)	–	–	1 (14.3)
Neutrophil count decreased	1 (9.1)	6 (54.5)	–	3 (42.9)
Lymphocyte count decreased	2 (18.2)	–	–	–
Lymphocyte count increased	1 (9.1)	–	–	–
*Electrolyte/laboratory abnormalities*				
Hyperbilirubinemia	1 (9.1)	–	1 (14.3)	–
Hypokalemia	–	–	4 (57.1)	–
Hypoalbuminemia	–	–	1 (14.3)	–
Hypomagnesemia	–	–	2 (28.6)	–
Creatinine increased	1 (9.1)	–	1 (14.3)	–
Hyperphosphatemia	–	–	1 (14.3)	–
Alanine aminotransferase increased	1 (9.1)	–	–	–
*Neurologic*				
Dizziness	1 (9.1)	–	1 (14.3)	–
Dysgeusia	–	–	1 (14.3)	–
Peripheral sensory neuropathy	3 (27.3)	–	1 (14.3)	–
*Cardiac*				
Electrocardiogram QT corrected interval prolonged	–	–	–	2 (28.6)
Atrial fibrillation	–	–	–	1 (14.3)
Palpitations	–	–	1 (14.3)	–
Hypertension	1 (9.1)	–	–	–
Sinus tachycardia	–	1 (9.1)	–	–
*Other*				
Fatigue	–	–	–	1 (14.3)
Flushing	1 (9.1)	–	–	–
Bone pain	1 (9.1)	–	–	–
Hypoxia	–	–	–	2 (28.6)
Hypotension	1 (9.1)	–	1 (14.3)	2 (28.6)
Sepsis	–	–	–	1 (14.3)
Edema limbs	–	–	1 (14.3)	–
Dyspnea	–	1 (9.1)	–	–
Infusion related reaction	1 (9.1)	–	–	–
Rash maculopapular	1 (9.1)	–	–	–
Folliculitis	–	–	1 (14.3)	–

Highest-grade adverse event per patient

^a^Port site infection

## Bidirectional Paclitaxel and Nilotinib

### Patient Characteristics

Seven patients were enrolled from October 13, 2022, through November 4, 2024, in a phase II, single-arm, open-label clinical trial investigating the efficacy of bidirectional paclitaxel plus nilotinib for treatment of peritoneal carcinomatosis from appendiceal (n=3), small bowel (n=1), or gastric cancers (n=3) with primary tumor sites in the body and fundus of the stomach (Table 1). Majority of participants were male (86%; 5/7), white (71%; 5/7), with a median age of 59 years (range 46–69), and median Eastern Cooperative Oncology Group performance status of 1 (range 0–2) at study enrollment (Table 1). Three patients (43%) had gastroesophageal reflux disease (GERD) that required medication changes prior to treatment initiation because of potential drug–drug interactions. One patient was on parenteral nutrition (PN) at study enrollment, and 43% (3/7) were initiated on PN after starting bidirectional paclitaxel plus nilotinib. All enrolled patients received prior systemic chemotherapy, with a median of two (range 1–4) lines of therapy per patient (Table 2). Two patients had measurable disease by RECIST 1.1 criteria prior to enrollment.^26^ The median baseline PCI obtained during screening laparoscopy upon study enrollment was 16 (range 13–24).

### Clinical Outcomes and Survival Characteristics

**Fig. 4:**
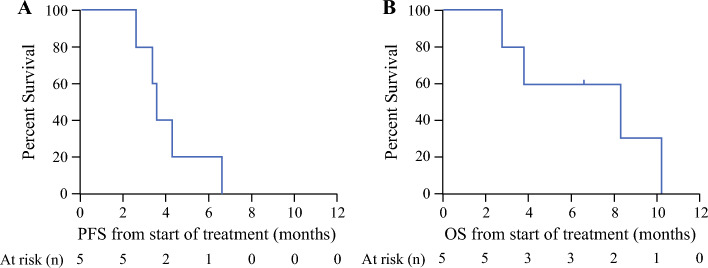
Kaplan–Meier curves for (A) clinical progression-free survival (PFS) and (B) overall survival (OS) from start of intraperitoneal/intravenous paclitaxel plus nilotinib treatment in months

Two patients were taken off study within 1 month of enrollment; one patient was unable to tolerate the leading dose of nilotinib and was taken off study before IP catheter placement, and a second patient developed sterile peritonitis requiring intensive care unit admission after receipt of the first dose of IP paclitaxel and was taken off study with subsequent removal of the IP catheter. Of the remaining five participants, median PCI at time of IP catheter placement was 20 (range 13–26), and median PCI upon completion of the first treatment course (cycles 1–3) was 16 (range 9–20). Only one (20%) of five participants was able to complete cycle 6 of bidirectional treatment, with a PCI increase of 3 points during the study period (PCI increased from 16 at trial enrollment to 19 at time of second re-staging laparoscopy). The median number of cycles completed among all five patients was 4 (range 3–7). Despite changes in PCI scoring, no patients receiving bidirectional paclitaxel and nilotinib met the primary endpoint of tumor downstaging to the point of resectability. During the study period, most (80%; 4/5) patients receiving IP/IV paclitaxel and nilotinib had stable disease, defined as PCI within 3 of baseline, and one patient exhibited an objective response to treatment, with PCI reduction from 15 to 9 after completion of cycles 1-3 (Table 2). One patient was taken off study after 5.8 months of treatment because they developed an abdominal abscess potentially worsened by prolonged neutropenia. Death due to disease progression occurred in one patient, and another patient died secondary to the sequelae of bacterial peritonitis after receiving cycle 4 IP/IV paclitaxel infusions. Two participants exhibited disease progression after 3.6 and 6.6 months. Overall median PFS was 3.6 months (range 2.6–6.6), and median OS was 8.3 months (range 2.8–10.2) for those receiving IP/IV paclitaxel plus nilotinib (Fig. 4A–B). There was also no evidence of objective histopathologic response to bidirectional paclitaxel and nilotinib therapy upon review of peritoneal biopsies obtained during laparoscopies by expert gastrointestinal pathologists. No patients developed extra-peritoneal disease progression during the study period. One patient went on to palliative bilateral oophorectomy after cycle 5, which constituted her radiographically apparent disease; she remained radiographically without evidence of disease at 6 months postoperatively.

### Safety and Morbidity

The most frequently reported grade 3–5 AEs were neutropenia (42.9%; 3/7), electrocardiogram QT prolongation (28.6%; 2/7), anemia (28.6%; 2/7), and hypoxia (28.6%; 2/7). Hypokalemia (57.1%; 4/7) was the most common grade 1–2 AE reported, followed by thromboembolic event (28.6%; 2/7), vomiting (28.6%; 2/7), neutropenia (28.6%; 2/7), and hypomagnesemia (28.6%; 2/7) (Table 3). All-cause mortality in participants who completed at least one cycle of IP/IV paclitaxel plus nilotinib was 80% (4/5) due to disease.

## Discussion

In this study, we reported the results of two small, heterogenous phase II, non-randomized, single-arm clinical trials investigating the bidirectional paclitaxel plus capecitabine or nilotinib for treatment of peritoneal-only and/or metastatic gastric, appendiceal, and small bowel adenocarcinoma. Bidirectional paclitaxel plus capecitabine therapy in gastric adenocarcinoma resulted in a median PFS of 5.3 months (95% CI 1.5–13.3) and median OS of 12.5 months (95% CI 4.7–14.7). Individuals receiving bidirectional paclitaxel plus nilotinib exhibited a median PFS of 3.6 months (range 2.6–6.6) and median OS of 8.3 months (range 2.8–10.2). Of note, stable disease was observed during the study periods in 64% and 80% of patients receiving bidirectional paclitaxel plus capecitabine or nilotinib, respectively. There was no evidence of extra-peritoneal disease progression during the study period for either bidirectional chemotherapeutic regimen. However, the small study size of both trials limited the statistical power of our findings. AEs were also common, and almost all participants in both trials experienced at least one grade 3–5 AE.

We demonstrated that the median OS of participants receiving bidirectional paclitaxel plus capecitabine was similar to those with alternative, more invasive cancer therapeutic strategies for advanced gastric cancer such as CRS with or without HIPEC.^16,27^ Peritoneal-only metastatic gastric adenocarcinoma treated with CRS plus HIPEC resulted in a median OS of 14.4–18.8 months compared with 12.5 months in the current study.^16,27^ More recent clinical trials demonstrated no improvement of OS with combination CRS plus HIPEC (OS 14.9 months; 97.2% CI 8.7–17.7) versus CRS alone (OS 14.9 months; 97.2% CI 7–19.4) for peritoneal-only metastatic gastric cancer.^28^ Furthermore, the role of CRS with or without HIPEC for metastatic gastric cancer remains controversial, sparking interest in alternative, less invasive locoregional therapeutic strategies such as bidirectional chemotherapy.

Improvement in OS with the addition of IP paclitaxel to systemic paclitaxel has been demonstrated in advanced ovarian cancer^29^; however, the efficacy of bidirectional paclitaxel in gastric cancer remains understudied. ^30^ Yonemura et al.^31^ found that neoadjuvant IP chemotherapy (docetaxel plus carboplatin) plus systemic chemotherapy resulted in disease downstaging and resection (49%; 30/61) in individuals with peritoneal-only metastatic gastric cancer. Improvement in PCI score after administration of IP paclitaxel has also been shown in peritoneal metastases in colorectal cancer.^32^ Ishigami et al.^30^ found no superiority of IP paclitaxel plus systemic chemotherapy compared with systemic chemotherapy alone in 183 patients with gastric cancer peritoneal metastases. However, bidirectional paclitaxel improved OS compared with systemic chemotherapy alone in a cohort of 222 patients with gastric cancer with peritoneal metastasis.^33^ In this study, we demonstrated that bidirectional paclitaxel plus capecitabine resulted in stable disease in most patients, suggesting a potential mechanism for delayed disease progression in individuals with peritoneal-only metastatic gastric adenocarcinoma. Similar to previous reports, we demonstrated bidirectional (IP/IV) paclitaxel therapy was well-tolerated. Chatterjee et al.^34^ found similar disease stability and tolerance of escalated doses of IP paclitaxel in patients with peritoneal-only metastatic colorectal cancer. Our results suggest that bidirectional paclitaxel plus capecitabine is a well-tolerated and feasible treatment strategy for patients with peritoneal-only metastatic gastric cancer.

Synergistic efficacy of oral nilotinib and IV paclitaxel was demonstrated in xenograft models for breast, renal, and ovarian cancer by the NCI-ALMANAC (A Large Matrix of Anti-Neoplastic Agent Combinations) study, providing the foundation for testing bidirectional paclitaxel and nilotinib.^35^ Despite promising preclinical data supporting synergistic efficacy of nilotinib and paclitaxel,^35,36^ the translational efficacy of this combination could not be determined in our study (NCT05185947) possibly because of (1) suboptimal dosing of the treatment combination due to uncontrolled concomitant medical conditions from discontinuation of effective supportive medications for potential drug–drug interactions with trial initiation, (2) late stage disease with locally advanced primary tumors and extensive small bowel involvement requiring total PN support during the trial, and (3) poor overall accrual due to overall disease rarity and the limited number of individuals with peritoneal-only metastatic disease, which ultimately led to the early trial closure. Poor gastrointestinal tolerability of this regimen was observed, albeit not manifested as AEs, leading to treatment holds, dose reductions, or study discontinuation. These patients underwent limited treatment exposure, with two patients coming off treatment before completing the first cycle and the remaining five patients receiving only a median of 3 cycles (range 2–6), which may have precluded potential responses to the combination study regimen with further dosing. The intolerability in this particular population may possibly be attributed to known nilotinib gastrointestinal adverse effects that might be more pronounced in the setting of advanced gastric primary involvement, diffuse peritoneal carcinomatosis and its sequelae, and/or global gastrointestinal dysmotility, poor nutritional status of the participants (four patients required PN for nutritional support), along with IP paclitaxel, as this is not noted in the phase I trial of IV paclitaxel and nilotinib trial or two other ongoing phase II trials of IV paclitaxel and nilotinib (^23^, personal communication with principal investigators). Additionally, prior history of GERD and change in GERD management due to potential drug interactions with nilotinib may have contributed to gastrointestinal symptoms (i.e. nausea, vomiting, and abdominal pain) even after dose reductions were implemented.

These two clinical trials illustrate that IP and systemic paclitaxel appear to be generally well-tolerated, and the bidirectional paclitaxel approach to treating peritoneal carcinomatosis (from gastric and gastroesophageal junction cancers) is currently being studied prospectively in the phase II STOPGAP trial.^37^ The results from our two single-arm clinical trials suggest that bidirectional paclitaxel may control systemic spread of tumor in patients with peritoneal carcinomatosis. Future studies combining IP/IV paclitaxel should explore other systemic agents that remain bioavailable in the peritoneum and are better tolerated in the setting of peritoneal carcinomatosis. Additionally, pre-clinical xenograft models for testing future oral agents in combination with paclitaxel should include peritoneal carcinomatosis tumor models, as well as IP administration (vs IV administration alone) of the intended bidirectional agent.

Our analyses were limited by small sample sizes and heterogeneity of primary tumor histologies. In both trials, patients received diverse systemic chemotherapy regimens prior to trial enrollment and had a wide range of baseline peritoneal disease burden (as quantified by PCI scores). Additionally, an inherent challenge of studying treatment effect on peritoneal carcinomatosis is the inability of even a highly experienced pathologist to objectively measure pathologic response to therapy based on non-blinded, heterogeneous peritoneal biopsies. Furthermore, intra-abdominal surgical scar from repeat staging laparoscopy may be indiscernible from post-treatment effect on tumor nodules. There is an unmet need for the development of specific and objective markers of treatment effect and peritoneal disease response in peritoneal malignancies. Future studies are warranted to explore the pharmacokinetics of tumor drug levels in peritoneal biopsies and ascites over treatment cycles.

## Conclusions

Both trials described here are noteworthy for their unique bidirectional therapy design, advancing our knowledge of potential management strategies for peritoneal carcinomatosis. Bidirectional paclitaxel-based chemotherapy prevented systemic progression and, in most patients, stabilized their peritoneal disease (especially in those receiving capecitabine). While these initial results appear promising, future, larger clinical trials are needed to more thoroughly investigate the efficacy of bidirectional therapy for peritoneal carcinomatosis. Ultimately, aiming for reduction of disease burden to subsequently enable cytoreduction should remain the goal as opposed to stability of disease. When designing these trials, identification of oral agents that are well-tolerated by patients with peritoneal carcinomatosis will be paramount.
